# Pharmacogenetics of dipeptidyl peptidase 4 inhibitors in a Taiwanese population with type 2 diabetes

**DOI:** 10.18632/oncotarget.14951

**Published:** 2017-02-01

**Authors:** Wen-Ling Liao, Wen-Jane Lee, Ching-Chu Chen, Chieh Hsiang Lu, Chien-Hsiun Chen, Yi-Chun Chou, I-Te Lee, Wayne H-H Sheu, Jer-Yuarn Wu, Chi-Fan Yang, Chung-Hsing Wang, Fuu-Jen Tsai

**Affiliations:** ^1^ Graduate Institute of Integrated Medicine, China Medical University, Taichung, Taiwan; ^2^ Center for Personalized Medicine, China Medical University Hospital, Taichung, Taiwan; ^3^ Department of Medical Research, Taichung Veterans General Hospital, Taichung, Taiwan; ^4^ Division of Endocrinology and Metabolism, Department of Medicine, China Medical University Hospital, Taichung, Taiwan; ^5^ School of Chinese Medicine, China Medical University, Taichung, Taiwan; ^6^ Department of Internal Medicine, Ditmanson Medical Foundation, Chiayi Christian Hospital, Chiayi City, Taiwan; ^7^ Department of Nursing, College of Nursing and Health Sciences, DAYEH University, Changhua, Taiwan; ^8^ Department of Business management, College of Management, National Chung Cheng University, Chia-yi, Taiwan; ^9^ National Center for Genome Medicine, Institute of Biomedical Sciences, Academia Sinica, Taipei, Taiwan; ^10^ Division of Endocrinology and Metabolism, Department of Internal Medicine, Taichung Veterans Hospital, Taichung, Taiwan; ^11^ School of Medicine, Chung Shan Medical University, Taichung, Taiwan; ^12^ School of Medicine, National Yang-Ming University, Taipei, Taiwan; ^13^ School of Medicine, National Defense Medical Center, Taipei, Taiwan; ^14^ Institute of Medical Technology, National Chung-Hsing University, Taichung, Taiwan; ^15^ Department of Genetics and Metabolism, Children's Hospital of China Medical University, Taichung, Taiwan; ^16^ School of Medicine, China Medical University, Taichung, Taiwan; ^17^ Department of Medical Genetics, China Medical University Hospital, Taichung, Taiwan; ^18^ Department of Health and Nutrition Biotechnology, Asia University, Taichung, Taiwan

**Keywords:** DPP-4 inhibitors, pharmacogenetics, Taiwanese, type 2 diabetes

## Abstract

Dipeptidyl peptidase-4 (DPP-4) inhibitors are oral anti-hyperglycemic drugs enabling effective glycemic control in type 2 diabetes (*T2D*). Despite DPP-4 inhibitors’ advantages, the patients’ therapeutic response varies. In this retrospective cohort study, 171 Taiwanese patients with T2D were classified as sensitive or resistant to treatment based on the mean change in HbA1c levels. Using an assumption-free genome-wide association study, 45 single nucleotide polymorphisms (SNPs) involved in the therapeutic response to DPP-4 inhibitors (*P* < 1 × 10^-4^) were identified at or near *PRKD1*, *CNTN3*, *ASK*, and *LOC10537792*. A SNP located within the fourth intron of *PRKD1* (rs57803087) was strongly associated with DPP-4 inhibitor response (*P* = 3.2 × 10^-6^). This is the first pharmacogenomics study on DPP-4 inhibitor treatment for diabetes in a Taiwanese population. Our data suggest that genes associated with β-cell function and apoptosis are involved in the therapeutic effect of DPP-4 inhibitors, even in the presence of additional oral anti-diabetic drugs. Our findings provide information on how genetic variants influence drug response and may benefit the development of personalized medicine.

## INTRODUCTION

Type 2 diabetes (T2D), also known as non-insulin-dependent diabetes mellitus, is a common complex disease with an increasing prevalence worldwide. In 2000, more than 171 million adults were diagnosed with T2D globally, which is expected to rise by 4.4%, affecting 366 million adults by 2030 [1].

The current treatment for T2D aims to control blood glucose levels and prevent diabetic complications. Nowadays, several anti-hyperglycemic drugs are available for T2D therapy, including metformin, sulfonylureas, thiazolidinediones (TZDs), alpha-glucosidase inhibitors, and insulin injections [2]. However, many patients with T2D experience initial success with anti-hyperglycemic drugs, only to become resistant to monotherapy over time, thus necessitating either an ancillary anti-diabetic agent or a transition to insulin in order to restore acceptable glycemic control. Approximately 40% of the individuals being treated for T2D fail to reach the desired glycosylated hemoglobin (HbA1c) target of <7%. In a study of long-term glycemic control in T2D, Kahn *et al*. [3] showed accumulative incidences of monotherapy failures at 5 years, including 15% failure with rosiglitazone (a TZD), 21% metformin (a biguanide), and 34% glyburide (a sulfonylurea). Thus, it is important to identify novel therapies that are more effective against T2D.

Dipeptidyl peptidase 4 (DPP-4) inhibitors are a class of oral hypoglycemic drugs approved by the FDA in 2006. Mechanistically, DPP-4 inhibitors increase incretin levels such as the levels of glucagon-like peptide-1 (GLP-1) and glucose-dependent insulinotropic polypeptides (GIP). GLP-1 and GIP are gut hormones secreted from L and K cells in the intestine in response to food intake [4, 5]. GLP-1 and GIP augment glucose-induced insulin release from pancreatic β-cells, suppress glucagon secretion, and slow gastric emptying [4, 5]. Both hormones are DPP-4 target proteins and are rapidly degraded and inactivated by proteolysis [6, 7]. Therefore, DPP-4 inhibitors, which can slow enzymatic cleavage that prevents the degradation of active incretins (GLP-1 and GIP), are used to enhance incretin-induced glycemic control. They have been proposed as potential therapeutic agents for T2D treatment.

DPP-4 inhibitors enable effective glycemic control with a low risk of hypoglycemia, neutral effects on body weight, and the convenience of once-daily oral dosing, which may improve patient adherence to therapy [8]. Several studies [9, 10] demonstrated that DPP-4 inhibitor treatment may be associated with a reduced incidence of cardiovascular (CV) events. Furthermore, DPP-4 inhibitors augment insulin secretion and increase β-cell mass by stimulating β-cell differentiation and proliferation by reducing oxidative and endoplasmic reticulum stress, inflammation, and apoptosis both *in vitro* and in pre-clinical models of T2D [11–15]. Recently, the DPP-4 inhibitors linagliptin [16] and saxagliptin [17] have been shown to improve glycemia and β-cell function in clinical trials. For these reasons, DPP-4 inhibitors are expected to improve treatment outcomes in patients with T2D.

A number of factors contribute to inter-individual differences in anti-diabetic drug responses, including age, sex, disease, drug and food interactions, co-morbidity, and genetic factors. A recent meta-analysis revealed that DPP-4 inhibitors decrease glycated hemoglobin (HbA1c) levels more markedly in Asians than in non-Asians [18], and the clinical factors underlying DPP-4 inhibitor resistance have been examined more intensely in Asian subjects [19–22]. Thus, genetic variations among different ethnic groups may alter the metabolism and therapeutic response of DPP-4 inhibitors, as previously demonstrated by pharmacogenomic and pharmacogenetic studies [23, 24]. Accordingly, the genetic effects of several genes such as *DPP4* [25, 26], *GLP1R* [27, 28], and *TCF7L2* [29] on the therapeutic response of DPP-4 inhibitors in patients with T2D have been investigated in clinical trial and case-control studies with a candidate gene approach. In the present report, we used an assumption-free genome-wide association study (GWAS) to identify the potential genes involved in the therapeutic response to DPP-4 inhibitors among patients with T2D in a Taiwanese population. While this is the first pharmacogenomic study of DPP-4 inhibitor treatment for diabetes in a Taiwanese population, the findings could provide some information on how genetic variants influence drug response and may benefit the development of personalized medicine.

## RESULTS

The study population consisted of 171 diabetic patients in stage I undergoing DPP-4 inhibitor therapy for GWAS. Four different DPP-4 inhibitors were prescribed, including sitagliptin, saxagliptin, vildagliptin, and linagliptin. The number of patients in each drug category was 114 (66.7%), 22 (12.9%), 23 (13.5%), and 12 (7%), respectively. Among them, 169 patients (98.9%) used DPP-4 inhibitors as a second treatment. Additionally, 29.8% and 64.3% of the patients were taking one or two oral anti-diabetic drugs (OADs), respectively, at the beginning of the study, and metformin and sulfonylurea were the two most common OADs used in the population. Detailed demographic and clinical characteristics of these patients are presented in Table 1. After DPP-4 inhibitor therapy, the patients exhibited significant differences in ΔHbA1c values, both with and without baseline HbA1c stratification (*p* < 0.001; data not shown). Further analysis revealed that the mean value of ΔHbA1c was significantly different among patients with different baseline HbA1c levels because patients with higher baselines (> 8%) displayed greater treatment responses (*p* < 0.001) (Table 2). Therefore, patients were classified as either sensitive or resistant based on their responses to DPP-4 inhibitor treatment (detailed information regarding the classification is provided in the Materials and Methods section). No significant association was observed between the response to DPP-4 inhibitor therapy and patient's sex, age, body mass index (BMI), disease duration, self-reported disease history (hypertension and cardiovascular disease), or lipid profile (cholesterol, triglycerides, high-density lipoprotein, and low-density lipoprotein) at enrollment (Table 3; all *p* > 0.05). However, the treatment-sensitive patients tended to have higher a BMI (> 27) than their resistant counterparts (44.8% vs. 31.2%; *p* = 0.052) (Table 3).

**Table 1:** Demographics of the study population in GWAS stage (stage I) for DPP-4 inhibitor pharmacogenomics study

	Total (N = 171)	Men (N = 80)	Women (N = 91)	P (♂vs♀)
**Age (years)**				
** ≦60 years (median)**	89 (52.0%)	43 (53.8%)	46 (50.5%)	
** >60 years**	82 (48.0%)	37 (46.3%)	45 (49.5%)	0.679
**BMI (kg/m^2^)**				
** <24**	49 (28.7%)	23 (34.8%)	26 (33.3%)	
** 24-27**	41 (24.0%)	20 (30.3%)	21 (26.9%)	
** >27**	54 (31.6%)	23 (34.8%)	31 (39.7%)	0.820
** Missing**	27 (15.8%)			
**eGFR**				
** ≧60 (median)**	116 (67.8%)	50 (75.8%)	66 (89.2%)	
** <60**	24 (14.0%)	16 (24.2%)	8 (10.8%)	0.035
** Missing**	31 (18.1%)			
**Baseline HbA1c (%)**				
** 7-<8%**	68 (39.8%)	35 (43.8%)	33 (36.3%)	
** 8-<9%**	55 (32.2%)	25 (31.3%)	30 (33.0%)	
** ≧9%**	48 (28.1%)	20 (25.0%)	28 (30.8%)	0.564
**Changed HbA1c (%)**	−0.95 ± 1.07	−0.98 ± 1.32	−0.92 ± 0.80	0.716
**DM duration (years)**	9.8 ± 6.5	9.32 ± 5.77	10.23 ± 7.07	0.361
**FPG (mg/dL)**	176.77 ± 47.53	171.38 ± 47.27	181.43 ± 47.53	0.172
**BP (mmHg)**				
** Systolic BP**	133.69 ± 15.61	135.32 ± 16.48	132.18 ± 14.79	0.213
** Diastolic BP**	80.34 ± 8.97	80.73 ± 9.01	79.97 ± 8.98	0.604
**OHA**				
** 1 OHA**	2 (1.2%)	2 (2.5%)	0 (0%)	
** 2 OHA**	51 (29.8%)	26 (32.5%)	25 (27.5%)	
** 3 OHA**	110 (64.3%)	49 (61.3%)	61 (67.0%)	
** 4 OHA**	8 (4.7%)	3 (3.8%)	5 (5.5%)	0.446

Values are presented as N (%) or mean ± SD; Abbreviation: BMI: body mass index; eGFR: estimated glomerular filtration rate; HbA1c: hemoglobin A1c; DMD: diabetes mellitus duration; FPG: fasting plasma glucose; BP: blood pressure; OHA: oral hypoglycemic agents. P value for two sample independent t test or ANOVA test

**Table 2:** The HbA1c difference from baseline to 3 months after enrollment

	N (%)	Changed HbA1cMean (SD)	P-value
**Sex**			
** Male**	80 (46.8%)	−0.984 (1.32)	0.716
** Female**	91 (53.2%)	−0.922(0.80)	
**Age**			
** ≦60**	89 (52.0%)	−0.988 (0.96)	0.641
** >60**	82 (48.0%)	−0.911 (1.18)	
**eGFR**			
** 50+**	128 (74.9%)	−0.907 (0.82)	
** <50**	12 (7.0%)	−0.458 (1.36)	0.092
**BMI**			
** <24**	49 (28.7%)	−1.137 (0.88)	
** 24-27**	41 (24.0%)	−0.866 (1.37)	
** >27**	54 (31.6%)	−0.813 (0.81)	0.243
**DM duration**			
** ≦8**	86 (50.3%)	−0.998 (0.98)	0.567
** >8**	85 (49.7%)	−0.904 (1.16)	
**Baseline HbA1c (%)**			
** 7-<8%**	68 (39.8%)	−0.465 (0.52)	<0.001
** 8-<9%**	55 (32.2%)	−0.918 (0.76)	
** 9%+**	48 (28.1%)	−1.677 (1.49)	

Abbreviation: BMI: body mass index; eGFR: estimated glomerular filtration rate; HbA1c: hemoglobin A1c; DM: diabetes mellitus; FPG: fasting plasma glucose; BP: blood pressure; OHA: oral hypoglycemic agents. P value for two sample independent t test or ANOVA test

**Table 3:** The baseline characters for sensitive groups and resistant groups

	Resistant Responders (N = 88)	Sensitive Responders (N = 83)	*P* value
**Sex**			
** Male**	44 (50%)	36 (43.4%)	
** Female**	44 (50%)	47 (56.6%)	0.385
**Age**			
** ≦60**	51 (58%)	38 (45.8%)	
** >60**	37 (42%)	45 (54.2%)	0.111
**BMI**			
** <24**	33 (42.9%)	16 (23.9%)	
** 24-27**	20 (26.0%)	21 (31.3%)	
** >27**	24 (31.2%)	30 (44.8%)	0.052
**eGFR**			
** ≧50**	66 (91.7%)	62 (91.2%)	
** <50**	6 (8.3)	6 (8.8%)	0.918
**DM duration**			
** ≦8**	50 (56.8%)	36 (43.4%)	
** >8**	38 (43.2%)	47 (56.6%)	0.079
**Baseline HbA1c**			
** 7-<8%**	35 (39.8%)	33 (39.8%)	
** 8-<9%**	31 (35.2%)	24 (28.9%)	
** 9%+**	22 (25%)	26 (31.3%)	0.566
Hypertension*			
** yes**	33 (42.9%)	35 (47.3%)	0.584
** no**	44 (57.1%)	39 (52.7%)	
Cardiovascular disease*			
** yes**	4 (8.3%)	7 (16.3%)	0.246
** no**	44 (91.7%)	36 (83.7%)	
**Cholesterol**	169.44 (29.51)	172.38 (29.47)	0.534
**Triglyceride**	149.06 (104.43)	166.75 (144.39)	0.384
**HDL**	49.25 (12.78)	47.30 (12.82)	0.344
**LDL**	99.25 (28.07)	99.65 (29.93)	0.933

Values are presented as N (%); Abbreviation: BMI: body mass index; eGFR: estimated glomerular filtration rate; HbA1c: hemoglobin A1c; DM: diabetes mellitus; HDL: high density lipoprotein; LDL: low density lipoprotein

*self-reported disease history P value for chi-square test.

In stage I, a preliminary GWAS, performed in 83 sensitive and 88 resistant patients, identified 45 single-nucleotide polymorphisms (SNPs) associated with the DPP-4 inhibitor treatment response (*p* < 10^−4^). A SNP located within the fourth intron of the protein kinase D1 gene (*PRKD1*; rs57803087) on chromosome 14 showed the strongest association with the DPP-4 inhibitor response (*p*-value for the trend test = 3.2 × 10^−6^) (Table 4). Two other SNPs, rs10511037 and rs62266510, located on chromosome 3 were also associated with the treatment response and are approximately 5-kb upstream of the contactin 3 gene (*CNTN3*). Chromosome 6 contained two pairs of significant SNPs, rs7755097/rs9376211 and rs4946688/rs1948999, located in the intron region of the gene encoding apoptosis signal-regulating kinase 1 (*ASK1*) and within an uncharacterized gene, *LOC105377923*, respectively. After adjusting for BMI as a potential confounding factor, rs57803087 on chromosome 14 continued to be the most predictive of the DPP-4 treatment response, with the other SNPs also maintaining their statistical significance.

**Table 4:** Summary of the SNPs associated with the effects of DPP-4 inhibitors in Type 2 diabetes in the GWAS stage and replication stage

dbSNP ID	Chr.	Nearest Gene	GWAS		Replication		Overall			
			Resistant Responders (n=88)	SensitiveResponders (n=83)	Resistant Responders (n=39)	Sensitive Responders (n=39)	Resistant Responders (n=127)	Sensitive Responders (n=122)	P value	Power
rs10511037	3	CNTN3	12.5%	31.5%	26.9%	28.2%	16.9%	30.5%	1.14E-06	70.6%
rs62266510	3	CNTN3	15.9%	33.9%	32.1%	34.6%	20.9%	34.1%	2.94E-07	63.3%
rs4946688	6	LOC105377923	54.5%	30.9%	46.2%	38.5%	52.0%	33.3%	3.69E-16	82.3%
rs1948999	6	LOC105377923	54.5%	31.3%	44.9%	37.1%	51.6%	33.2%	7.89E-16	81.1%
rs7755097	6	ASK1	12.5%	30.1%	17.9%	21.8%	14.2%	27.5%	3.49E-06	71.1%
rs9376211	6	ASK1	19.8%	40.7%	-	-	-	-	-	-
rs57803087	14	PRKD1	17.0%	38.6%	19.2%	26.9%	17.7%	34.8%	2.51E-08	86.5%

Abbreviation: dbSNP ID: SNP database identification; Chr: chromosome; GWAS

*Risk allele is the allele with higher frequency

Subsequently, we replicated the six most significant SNPs from stage I in an additional 39 sensitive responders and 39 resistant responders. None of the SNPs showed a significant association with the DPP-4 inhibitor response. After the GWAS (stage I) and replication (stage II) results were combined, rs57803087, located within *PRKD1*, remained significantly associated with the DPP-4 inhibitor response (Table 4).

## DISCUSSION

To our knowledge, this is the first pharmacogenomic study of DPP-4 inhibitor treatment for T2D in a Taiwanese population. In the present study, DPP-4 inhibitors served as an add-on therapy. More than 98% of the subjects used more than one OAD instead of DPP-4 inhibitors alone, and the average HbA1c level was reduced by 0.95%, which is comparable with the data from a previous report [9]. Several factors can influence the therapeutic response to DPP-4 inhibitors as a primary or ancillary treatment [19, 30]. Baseline HbA1c values and shorter disease durations are important predictors of the efficacy of OADs, including DPP-4 inhibitors [8]. In our retrospective cohort study, subjects with higher baseline HbA1c values showed a better therapeutic response to DPP-4 inhibitor treatment. However, no significant association with disease duration was observed.

DPP-4 inhibitors prevent the degradation of incretins, GLP-1 and GIP, in response to glucose-dependent insulin secretion. Previous studies showed that polymorphism in *DPP4* [25, 26] and *GLP1R* [27, 28], which are directly involved in the mechanism of action of DPP-4 inhibitors, was associated with the glycemic response to DPP-4 inhibitor treatment. Appropriate regulation of insulin secretion may also be related to DPP-4 inhibitor efficacy. Our data indicate that genes associated with β-cell function and apoptosis may be involved in the therapeutic effect of DPP-4 inhibitors, even when additional OADs are used. First, rs9376211 is located withinthe *ASK1* intron region on chromosome 6. Previous studies have shown that *ASK1* variants are associated with skeletal muscle ASK1 expression, *in vivo* insulin resistance, and T2D in Pima Indians [31]. *ASK1* encodes the ASK1 protein, a member of the large mitogen-activated protein kinase kinase kinase (MAP3K) family that activates downstream MAPKs, c-Jun N-terminal kinase (JNK) and p38 MAPK, and is essential for the cellular response to various stressors. As such, ASK1 signaling can elicit cell death, differentiation, inflammation, and survival [32, 33]. In pancreatic β-cells, ASK1 is involved in the oxidative stress-induced thioredoxin-interacting protein (TXNIP)-dependent apoptosis cascade [32, 34]. Moreover, recent studies have demonstrated that the TXNIP signaling pathway is involved in inhibition of β-cell apoptosis by GIP [35, 36]. Collectively, our data suggest that the increase in GIP concentration mediated by DPP-4 inhibitors may inhibit pancreatic β-cell death. Thus, patients harboring ASK1 variants, in whom GIP-mediated cell protection is compromised, may be more resistant to this form of therapy.

A second SNP (rs57803087) was located in the intron region of the *PRKD1* gene on chromosome 14. PRKD1 is a serine/threonine kinase that controls a variety of cellular functions, including membrane receptor signaling, Golgi transport, mitochondrial oxidative stress responses, gene transcription, cell morphology, motility, and adhesion [37]. Studies have demonstrated that agonists of G-protein-coupled receptor 40 (GPR40) and free fatty acid (FFA) receptor 1 (FFAR1) are induced by β-cell insulin secretion [38]. Additionally, PRKD1 activation contributes to the GPR40-mediated insulin secretion from β-cells [39]. FFA-induced GPR40 activation results in the generation of diacyl glycerol via the phospholipase C-mediated hydrolysis of membrane phospholipids, PRKD1 activation, cortical actin depolymerization, and potentiation of a second phase of glucose-stimulated insulin secretion [39]. Moreover, Kong *et al*. [40] have reported that the M3 muscarinic receptor promotes the insulin release via receptor phosphorylation/arrestin-dependent PRKD1 activation. Importantly, both of these PRKD1 activation mechanisms potentiate insulin secretion.

There are several limitations to this study. First, we recognize that this study had low statistical power, owing to its limited sample size. Based on the sample size (127 resistant responders vs. 122 sensitive responders after combining the subjects from the stage I GWAS and stage II replication) and the difference in the allele frequency for the revealed SNPs (range: from 13.2% to 18.7%), the power was 63.3% to 86.5% (Table 4). The results did not reach statistical significance after a Bonferroni correction, and false positive results may exist. Very little is known about the contribution of patient genetics to DPP-4 inhibitor responses. Therefore, analyses with larger sample sizes could potentially identify additional genetic variants to enable better predictions for personalized or stratified medicine. Second, metformin is known to increase GLP-1 secretion by intestinal L-cells. Thus, the combination of metformin and DPP-4 inhibitor treatment may exert a stronger effect on β-cell function [41, 42]. In the present study, >98.8% of patients received other drug therapies in addition to DPP-4 inhibitors. For this reason, the therapeutic effect of single-agent DPP-4 inhibitors could not be determined. Third, the insulin secretory capacity decreases according to the duration of T2D [43] and could thus be another predictor of the DPP-4 inhibitor efficacy [8, 19, 30]. Accordingly, clinical trials have shown that the DPP-4 inhibitors linagliptin [16] and saxagliptin [17] better preserved β-cell function in recently diagnosed (< 24 months) T2D patients. Similarly, the Saxagliptin Assessment of Vascular Outcomes Recorded in Patients with Diabetes Mellitus (SAVOR-TIMI 53) trial showed that saxagliptin-mediated preservation of β-cell function was better in patients with higher homeostatic model assessment 2 (HOMA2) scores prior to DPP-4 inhibitor treatment [17]. However, the baseline β-cell function was not measured in the present study. Thus, we cannot exclude the effect of variations in baseline β-cell function on the DPP-4 therapeutic response. Fourth, we were unable to exclude the possibility of drug interactions, which would need to be further investigated. For example, exposure to saxagliptin and its primary metabolite may be significantly modified when saxagliptin is co-administered with strong specific inhibitors (ketoconazole or diltiazem) or inducers (rifampicin) of the cytochrome P450 3A4/5 isoform [44]. Moreover, some lipid-lowering drugs such as statins could increase the HbA1c value. Unfortunately, the information on lipid-lowering drugs taken by the recruited patients simultaneously with DPP-4 drugs was not available. However, no significant differences in the hypertension history, cardiovascular disease history, or lipid profiles (cholesterol, triglycerides, low-density lipoprotein, and high-density lipoprotein) were observed between the responders and non-responders at enrollment; therefore, the potential for selection bias was minimal.

In conclusion, the current study utilized a GWAS-based approach to examine the pharmacogenetics of DPP-4 inhibitor treatment for T2D in a Taiwanese population. Our data indicate that genes associated with β-cell function and apoptosis may be involved in the therapeutic effect of DPP-4 inhibitors, even when additional OADs are used. However, further studies are required to confirm the association of the genes identified in this study with the therapeutic effect of DPP-4 inhibitors using a larger, more ethnically diverse patient population.

## MATERIALS AND METHODS

### Study population identification

This was a retrospective cohort study using data generated from a systematic chart review of diabetic patients from our previous study population [45, 46], treated with a consistent dosage of DPP-4 inhibitors for > 60 days. Endocrinologists performed the chart review. Co-treatment with other OADs was permitted if dosing was maintained for 3 months before and after DPP-4 inhibitor treatment. However, no incretin analog was allowed together with the DPP-4 inhibitors. Subjects with type 1 diabetes or undergoing insulin therapy were excluded from the analyses. Because DPP-4 inhibitors are primarily excreted in the urine and could be subject to renal function-associated effects, subjects with estimated glomerular filtration rates (eGFRs) of less than 30 mL/min per 1.73 m^2^ were excluded from the study. Additionally, patients with eGFRs between 30 and 50 mL/min per 1.73 m^2^, taking ≤ 50 mg of sitagliptin/vildagliptin or ≤ 2.5 mg of saxagliptin/linagliptin, were also excluded. The study was approved by the Institutional Review Board of the China Medical University Hospital, and informed consent was obtained from all participants.

The patients were first stratified according to their baseline HbA1c values (≤ 6.99%, 7–7.99%, 8–8.99%, and ≥ 9%) into four groups. The subjects were then classified as either sensitive or resistant to DPP-4 inhibitor treatment based on their treatment response change, determined by ΔHbA1 (the difference in HbA1c values before and after treatment). In each group, subjects with the ΔHbA1 values higher or lower than the mean ΔHbA1 value for the group were defined as sensitive or resistant, respectively. In this two-stage study, we first genotyped 83 sensitive responders and 88 resistant responders via an exploratory genome-wide scan (stage I, total 171 subjects). In the replication stage, we genotyped six selected SNPs in an additional 39 sensitive responders and 39 resistant responders (stage II, total 78 subjects).

### Genotyping and statistical analysis

Blood glucose and HbA1c levels were recorded before and > 60 days after a DPP-4 inhibitor treatment course. Genomic DNA from blood samples was genotyped with an Affymetrix CHB chip using standard quality control procedures. SNPs with the following conditions were excluded from the analysis: (1) individuals or SNPs with a call rate of < 95%; (2) *p* ≤ 0.0001 for the Hardy–Weinberg test for control; (3) SNPs with a minor allele frequency of < 0.01; (4) samples with first-degree cryptic relationships; and (5) samples that were potentially 9% contaminated. In total, 618,882 SNPs were included in the GWAS.

Various clinical variables were compared between groups using *χ*^2^ tests or two independent *t*-tests. The threshold *p*-value was set at 8.0 × 10^−8^ after a Bonferroni correction for SNP numbers (*n* = 618,882). All statistical analyses were conducted using the SAS statistical software, version 9.1 (SAS Institute, Inc., Cary, NC, USA). *p* < 0.05 (two-sided) was considered significant.
